# Renal Artery Pseudoaneurysm in Pregnancy Managed Successfully With Endovascular Embolisation

**DOI:** 10.7759/cureus.102525

**Published:** 2026-01-28

**Authors:** Anitha Reddy Depa, Rajitha Reddy, Vanaparthi Kavya

**Affiliations:** 1 Obstetrics and Gynaecology, Fernandez Hospital, Hyderabad, IND

**Keywords:** antenatal diagnosis, colour doppler ultrasound, endovascular embolization, pregnancy, renal artery aneurysm, renal artery pseudoaneurysm, vascular complications in pregnancy

## Abstract

Renal artery pseudoaneurysm is a rare but potentially life-threatening vascular condition, particularly when it occurs during pregnancy. Delayed recognition may lead to catastrophic haemorrhage with significant maternal and foetal morbidity. We present a case of a pregnant woman diagnosed with a renal artery pseudoaneurysm during the antenatal period who was successfully managed with endovascular embolisation. Prompt imaging, coordinated multidisciplinary care, and minimally invasive intervention enabled stabilisation of the condition and continuation of pregnancy, resulting in favourable maternal and neonatal outcomes. This case underscores the importance of early diagnosis and highlights endovascular therapy as a safe and effective treatment option during pregnancy.

## Introduction

Renal artery pseudoaneurysm is an exceptionally rare vascular condition in pregnancy [1], but it may be associated with significant maternal and foetal morbidity if not promptly recognised [2]. Physiological changes during pregnancy, including increased circulating blood volume, elevated cardiac output, and hormonally mediated vascular wall remodelling, contribute to an increased risk of aneurysmal expansion and rupture [3]. Historically, maternal mortality rates of up to 40-45% and foetal mortality approaching 20-25% have been reported in cases of rupture [1,4]. The natural history of renal artery aneurysms further suggests an increased susceptibility to rupture during pregnancy due to combined haemodynamic stress and hormonal influences [5]. Early diagnosis and timely multidisciplinary management are therefore essential to optimise maternal and foetal outcomes [6]. This case highlights the educational value of early antenatal diagnosis of a renal artery pseudoaneurysm prior to catastrophic rupture and its successful management using a minimally invasive endovascular approach, thereby contributing to the limited existing literature on optimal diagnostic and therapeutic strategies for this rare condition in pregnancy.

## Case presentation

A 27-year-old primigravida at 23 weeks' gestation presented with sudden-onset right-sided flank pain associated with nausea and vomiting. She had no history of trauma, hypertension, connective tissue disorder, or prior renal disease. On examination, she was haemodynamically stable with mild right renal angle tenderness. Laboratory evaluation revealed a significant drop in haemoglobin from 13.7 g/dL to 8.6 g/dL (Table 1). In a pregnant woman presenting with acute flank pain and anaemia, the initial differential diagnosis included more common conditions such as renal colic, acute pyelonephritis, placental abruption, ureteric obstruction, renal vein thrombosis, and musculoskeletal pain. Less frequent vascular aetiologies, including renal infarction and renal artery aneurysm or pseudoaneurysm, were also considered. The absence of fever, urinary symptoms, trauma, hypertension, and underlying renal or connective tissue disease, together with progressive anaemia, prompted further evaluation for a vascular aetiology.

**Table 1:** Investigations trend

Parameter	Timing	Patient values	Unit	Reference range (pregnancy)	Interpretation
Haemoglobin	Pre-embolisation (24/08/2025); Post-embolisation (26/08–31/08/2025)	8.6 → 8.1 → 8.6	g/dL	11.0–14.0	Moderate anaemia with a stable trend; no evidence of ongoing haemorrhage
Total leukocyte count	Pre & Post	10,770–15,980	/mm³	6,000–16,000	Leukocytosis consistent with an inflammatory response
Blood urea	Pre (24/08); Post (31/08)	17 → 8	mg/dL	7–18	Normal renal clearance
Serum creatinine	Pre & Post	0.5	mg/dL	0.4–0.8	Preserved renal function throughout
Sodium (Na⁺)	Pre & Post	134–136.1	mmol/L	135–145	Within an acceptable range
Potassium (K⁺)	Pre & Post	3.6–4.0	mmol/L	3.5–5.0	Normal
Chloride (Cl⁻)	Pre & Post	103–105	mmol/L	98–107	Normal
Prothrombin time	Pre-embolisation (24/08)	12	seconds	11–14	Normal coagulation profile
INR	Pre-embolisation (24/08)	0.87	-	<1.2	No coagulopathy
C-reactive protein	Post-embolisation (28/08–31/08)	176.5 → 192 → 146	mg/L	<5	Markedly elevated inflammatory marker with a declining trend post-intervention
Urine routine	Post-embolisation (26/08)	Glucose 3+, no haematuria	-	No glucose/haematuria	No urinary bleeding
Urine culture	Post-embolisation (27/08)	-	-	Sterile	Infection ruled out

**Figure 1:**
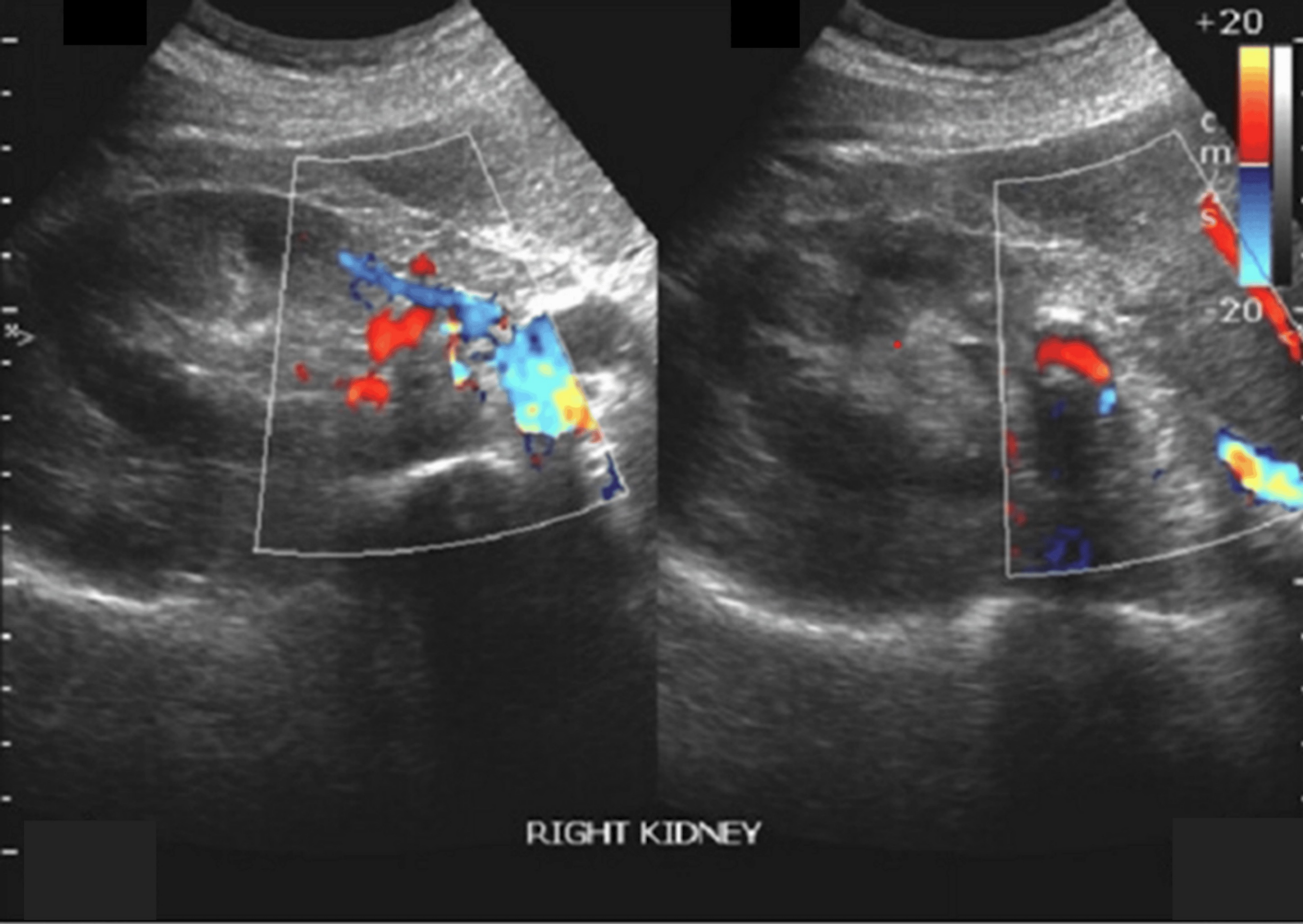
USG abdomen prompting further cross-sectional imaging Colour Doppler ultrasonography of the right kidney demonstrating a bulky kidney with a large perihilar vascular lesion showing turbulent high-velocity bidirectional flow (yin-yang appearance), suggestive of a renal artery pseudoaneurysm. The surrounding renal parenchyma appears compressed, with early calyceal dilatation. These findings raised suspicion of an underlying renal vascular abnormality, prompting further cross-sectional imaging.

Abdominal ultrasonography with colour Doppler demonstrated a bulky right kidney with a large perihilar vascular lesion exhibiting turbulent, high-velocity flow, raising suspicion of an underlying vascular abnormality (Figure 1).

**Figure 2:**
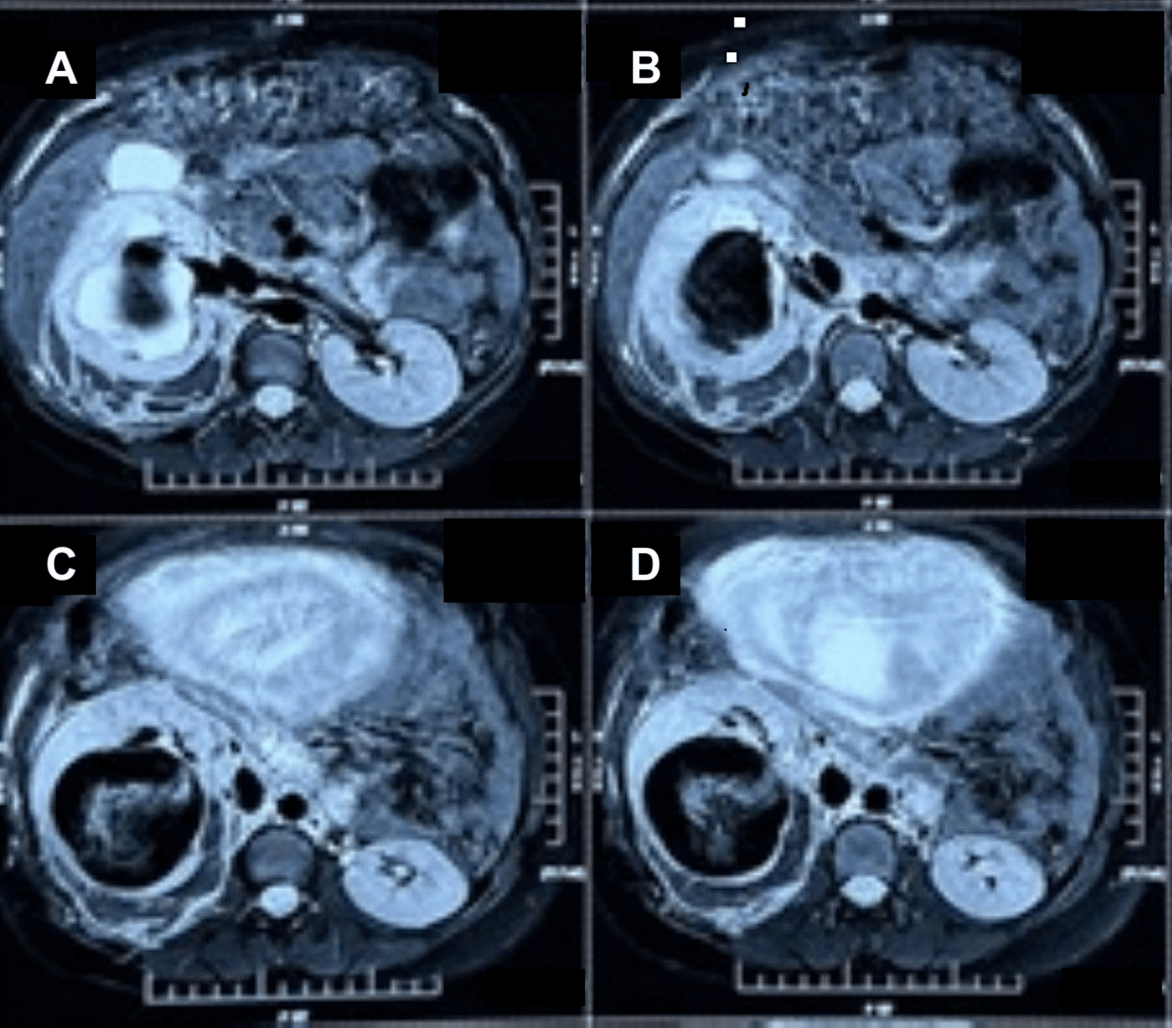
MRI abdomen (axial images) Axial magnetic resonance imaging of the abdomen demonstrating a left renal artery pseudoaneurysm with associated intrarenal haematoma. (A) Axial T2-weighted image showing a well-defined hyperintense lesion in the left renal hilum consistent with a pseudoaneurysm. (B) Axial T2-weighted image demonstrating the pseudoaneurysm with surrounding haematoma within the left kidney. (C) Axial T2-weighted image showing extension of the intrarenal haematoma with mass effect on adjacent renal parenchyma. (D) Axial T2-weighted image confirming the location of the lesion and associated intrarenal haematoma.

The woman was subsequently referred to a vascular surgeon for further evaluation, following which an MRI of the abdomen and a non-contrast renal angiogram were performed, confirming a renal artery pseudoaneurysm arising from an interlobar branch, with no evidence of renal malignancy or perinephric haematoma (Figure 2).

**Figure 3:**
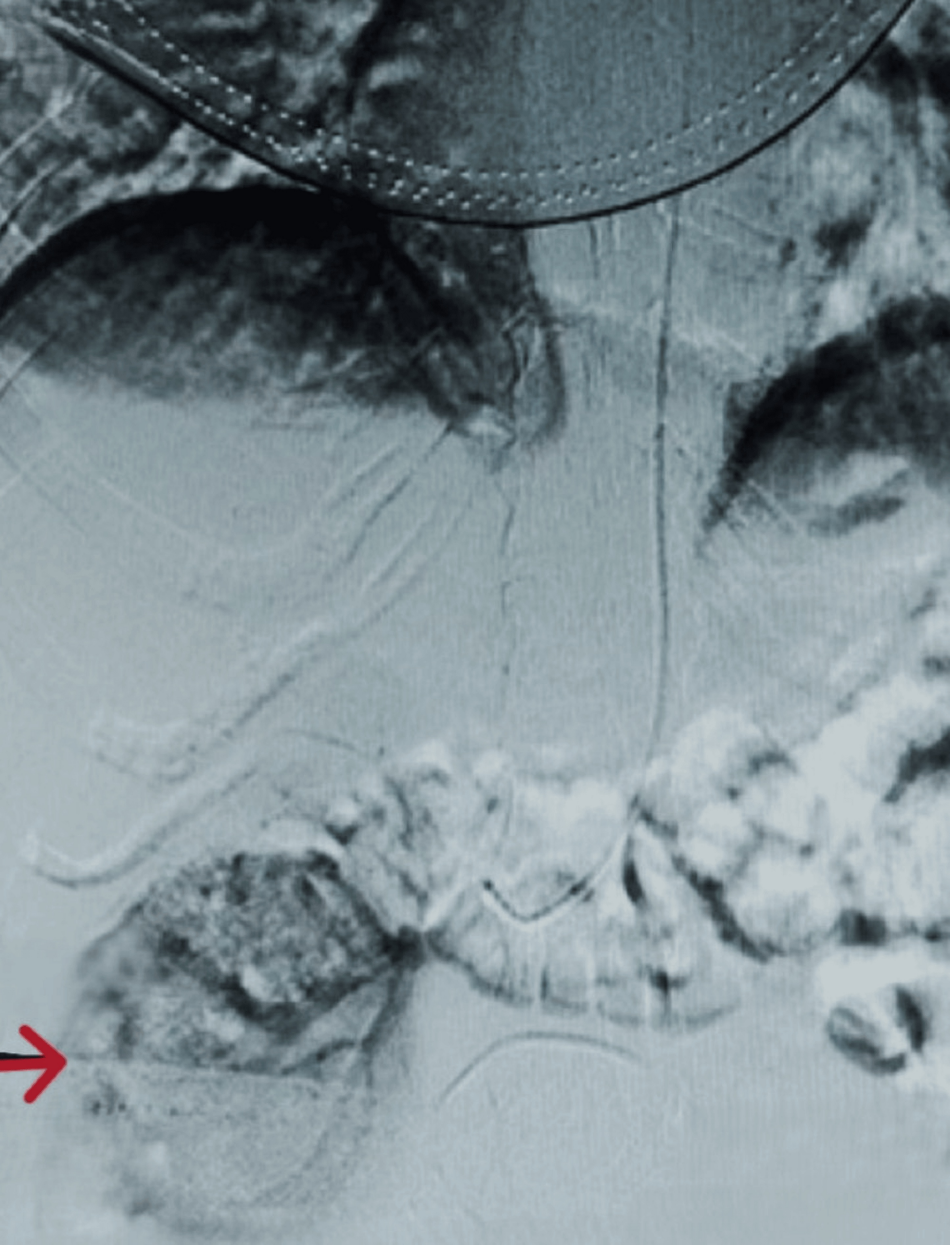
Selective right renal artery angiography and embolisation The red arrow highlights the pseudoaneurysmal sac, characterised by turbulent, high-velocity flow within the lesion, consistent with a renal artery pseudoaneurysm.

**Figure 4:**
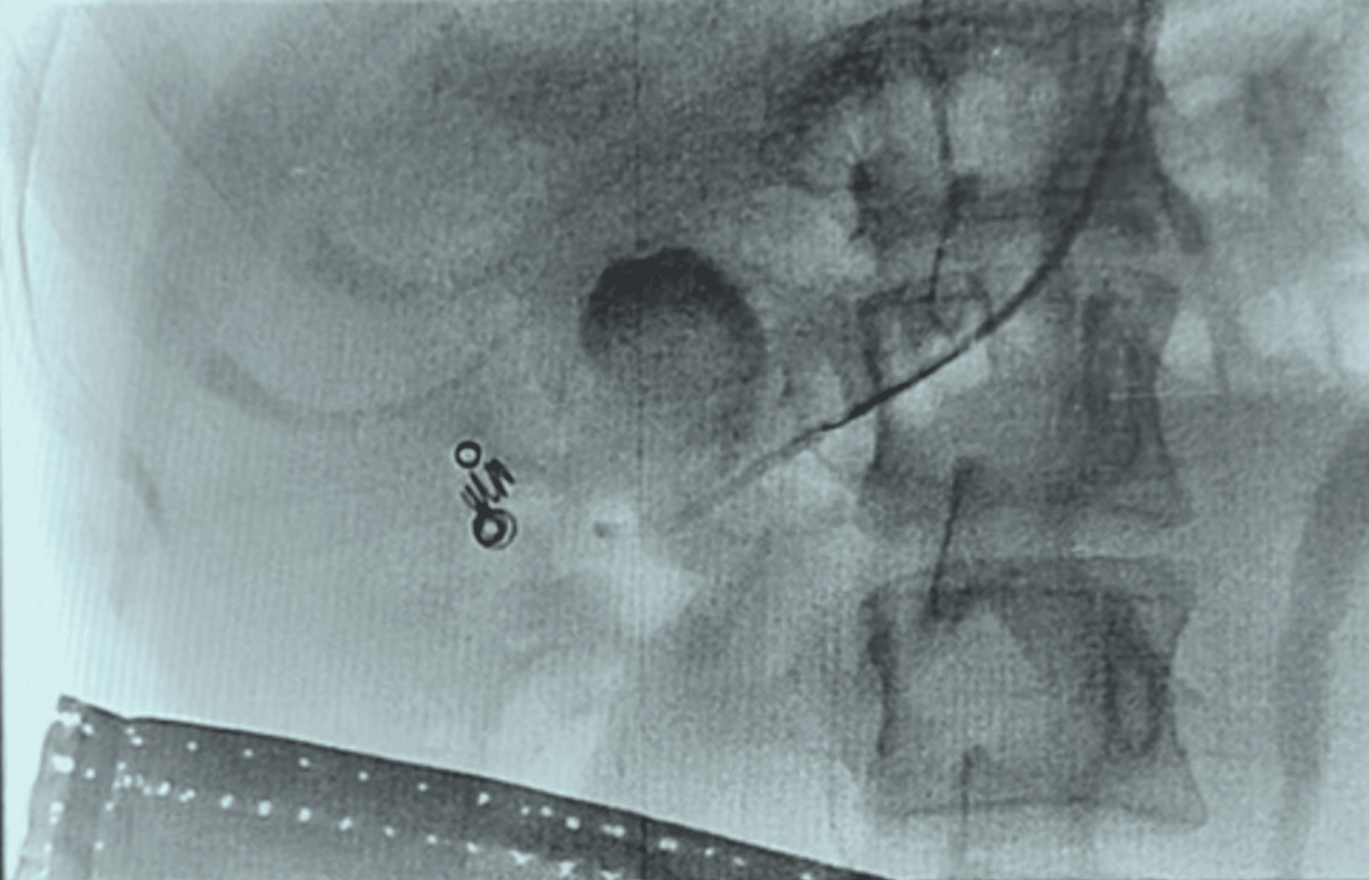
Deployment of embolisation coils within the feeding vessel

**Figure 5:**
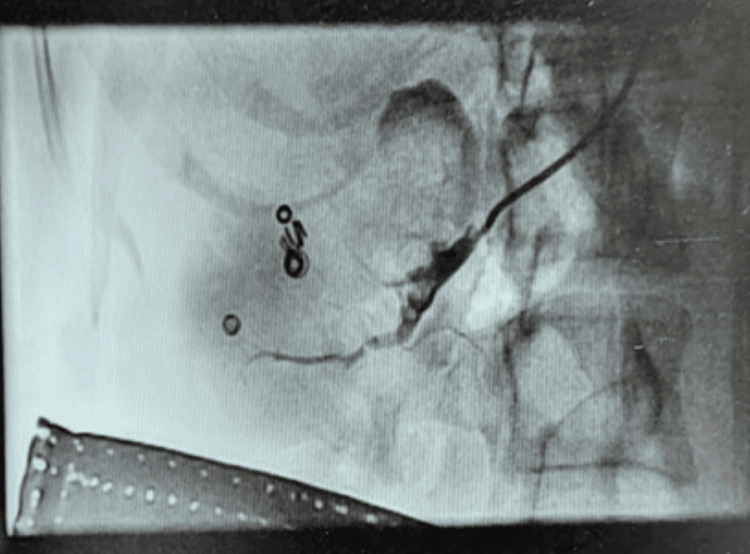
Post-embolisation angiogram showing complete exclusion of the pseudoaneurysm with no residual contrast filling

The couple was counselled in detail regarding the diagnosis, potential risks, available treatment options, and the benefits and limitations of endovascular management. After multidisciplinary discussion and obtaining informed written consent, the patient underwent successful endovascular embolisation under local anaesthesia (Figures 3-5). Notably, embolisation was performed using a combination of detachable coils and N-butyl cyanoacrylate glue, avoiding general anaesthesia and minimising foetal exposure, which is rarely reported in pregnancy-associated renal artery pseudoaneurysms. Vascular access was obtained via the left brachial artery using a 5-Fr sheath. The right renal artery was selectively cannulated, and a microcatheter was advanced super-selectively into the feeding segmental branch, allowing targeted embolisation with preservation of renal parenchyma.

Post-procedural course and follow-up

Following endovascular coil embolisation, the patient made an uneventful immediate post-procedural recovery. She was monitored in the medical intensive care unit, where serial haemoglobin levels remained stable with no evidence of ongoing bleeding. Renal function remained normal throughout her hospital stay.

She experienced a transient febrile episode, with a maximum recorded temperature of 102.1°F, which resolved with conservative management. Blood and urine cultures showed no growth, and inflammatory markers gradually trended down. She remained haemodynamically stable and was discharged in good condition.

**Figure 6:**
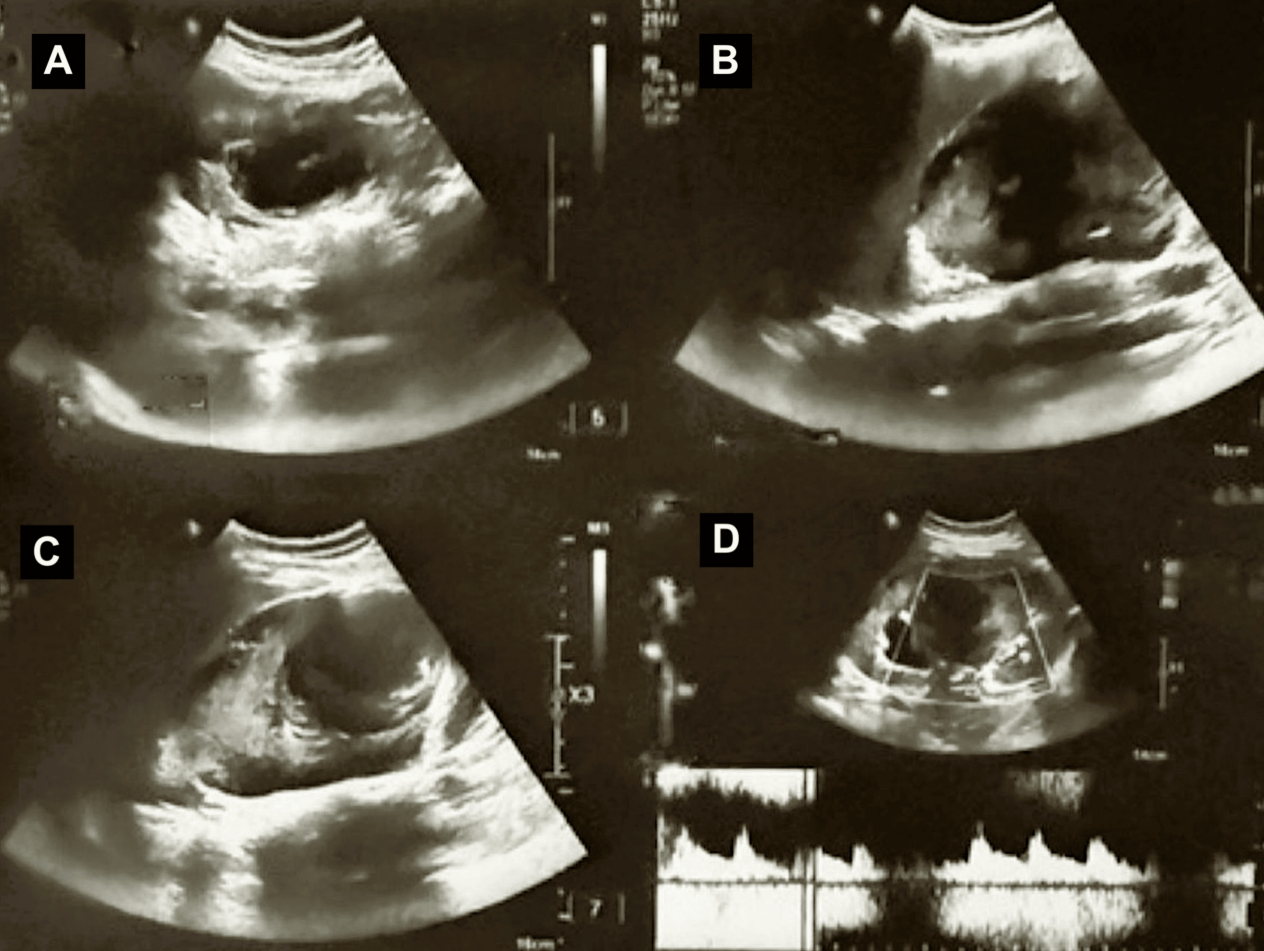
Post-procedure USG Post-embolisation ultrasound of the right kidney demonstrating a thrombosed renal artery pseudoaneurysm and associated subcapsular haematoma. (A) Long-axis greyscale ultrasound showing a bulky right kidney with a well-defined intrarenal lesion. (B) Transverse greyscale ultrasound demonstrating the thrombosed pseudoaneurysm with surrounding organised haematoma. (C) Greyscale ultrasound image showing the organised subcapsular haematoma adjacent to the kidney. (D) Colour and spectral Doppler images demonstrating the absence of internal vascular flow within the pseudoaneurysm and the preservation of intrarenal arterial flow following embolisation.

Serial ultrasonography during hospitalisation demonstrated a bulky right kidney with a thrombosed renal artery pseudoaneurysm causing moderate upper-pole calyceal dilatation, along with a right subcapsular collection consistent with an organised haematoma. These findings remained stable on interval imaging (Figure 6).

On outpatient follow-up, repeat imaging showed no increase in the size of the pseudoaneurysm, with progressive organisation of the haematoma. Renal Doppler studies did not demonstrate any haemodynamically significant renal artery stenosis. Clinically, the woman remained asymptomatic with normal blood pressure and preserved renal function. Antenatal follow-up thereafter was uneventful. Serial foetal assessments demonstrated appropriate growth and a normal amniotic fluid volume, while maternal anaemia improved with oral iron supplementation. At subsequent visits, she remained clinically stable without recurrence of abdominal pain, haematuria, or hypertension.

The pregnancy progressed without further complications. Although vaginal birth was discussed as a feasible option, the couple opted for an elective lower-segment caesarean section. She underwent a caesarean section at 38 weeks of gestation and delivered a healthy female infant weighing 3000 g. Both maternal and neonatal outcomes were satisfactory.

## Discussion

Renal artery pseudoaneurysm in pregnancy is an exceptionally rare vascular condition, but it is associated with significant maternal and foetal morbidity when rupture occurs. Pregnancy-related physiological changes, including increased circulating blood volume, elevated cardiac output, and hormonally mediated arterial wall remodelling, predispose to aneurysmal expansion and rupture [1,3]. Historically, maternal mortality rates of up to 40-45% and foetal mortality rates approaching 20-25% have been reported in cases of rupture, underscoring the importance of early diagnosis and timely intervention [1,4]. The natural history of renal artery aneurysms suggests an increased risk of rupture during pregnancy due to haemodynamic stress and hormonal influences [5].

Clinical presentation is often nonspecific, with flank or abdominal pain frequently mimicking more common obstetric or urological conditions such as renal colic, pyelonephritis, or placental abruption [3,7,8]. This diagnostic overlap contributes to delayed recognition in many reported cases, with diagnosis often made only after haemodynamic compromise. In contrast, early imaging in our patient enabled diagnosis prior to rupture, allowing a planned and controlled management strategy.

Imaging plays a pivotal role in diagnosis and surveillance. Ultrasonography with colour Doppler is typically the initial modality, enabling identification of renal enlargement and vascular lesions with turbulent flow. MRI without contrast provides superior soft-tissue characterisation while avoiding ionising radiation and gadolinium exposure, making it well-suited for use in pregnancy [3]. Renal angiography remains the diagnostic gold standard, providing precise vascular delineation and enabling simultaneous therapeutic intervention when required [2].

Management strategies depend on haemodynamic stability, gestational age, and available expertise. While open surgical repair or nephrectomy was historically common, endovascular techniques have increasingly emerged as the preferred approach in stable patients. Endovascular coil embolisation offers high technical success rates, preserves renal parenchyma, and avoids the morbidity associated with open surgery [6,9]. Several case reports and small series have demonstrated favourable maternal and foetal outcomes following endovascular embolisation during pregnancy [6,7,10].

Our case is notable for early diagnosis prior to catastrophic rupture, radiological stability on serial imaging, and successful endovascular management under local anaesthesia, resulting in preservation of renal function and favourable maternal and foetal outcomes. Contained rupture of renal artery pseudoaneurysms may present with subtle clinical signs and preserved haemodynamic stability due to local tamponade by an intraparenchymal haematoma, as demonstrated in our patient, thereby permitting planned definitive endovascular management. Similar outcomes have been reported in previously published cases managed with endovascular techniques [6,7,10]. This case highlights the importance of early recognition and multidisciplinary collaboration and supports endovascular embolisation as a safe and effective treatment option for renal artery pseudoaneurysm in pregnancy when appropriate expertise is available.

Learning points

Renal artery pseudoaneurysm, although rare, should be considered in pregnant patients presenting with unexplained flank or abdominal pain and anaemia. (1) Pregnancy-safe imaging modalities, including ultrasonography and MRI, enable early diagnosis and facilitate timely intervention before catastrophic rupture. (2) In haemodynamically stable patients, serial imaging can be safely used to assess lesion stability and guide the timing of definitive treatment. (3) Super-selective endovascular embolisation under local anaesthesia provides a kidney-preserving and minimally invasive treatment option with favourable maternal and foetal outcomes when performed by an experienced multidisciplinary team.

## Conclusions

Renal artery pseudoaneurysm in pregnancy is a rare but potentially life-threatening condition. Early recognition, prompt pregnancy-safe imaging, and close multidisciplinary collaboration are crucial to prevent catastrophic maternal and foetal complications. This case demonstrates that, in selected hemodynamically stable patients, planned super-selective endovascular embolisation under local anaesthesia can achieve definitive treatment with preservation of renal function and favourable maternal and foetal outcomes.
